# *Lactobacillus rhamnosus* ameliorates acne vulgaris in SD rats *via* changes in gut microbiota and associated tryptophan metabolism

**DOI:** 10.3389/fimmu.2023.1293048

**Published:** 2024-01-05

**Authors:** Yukun Huang, Yaxin Huang, Dengmei Xia, Lu Liu, Xia Xiong, Yongliang Ouyang, Yongqiong Deng

**Affiliations:** ^1^ Department of Dermatology, the Affiliated Hospital of Southwest Medical University, Luzhou, Sichuan, China; ^2^ Department of Burn and Plastic Surgery, Zigong Fourth People’s Hospital, Zigong, Sichuan, China; ^3^ Department of Health Management, Luzhou People’s Hospital, Luzhou, Sichuan, China; ^4^ Department of Dermatology, Chengdu First People’s Hospital, Chengdu, Sichuan, China

**Keywords:** *Lactobacillus rhamnosus*, acne vulgaris, gut microbiota, tryptophan, aryl hydrocarbon receptor

## Abstract

**Background:**

The depletion of beneficial bacteria in the gut has been found in patients with acne vulgaris, and in previous studies, the supplement of *Lactobacillus rhamnosus* led to the improvement of adult acne. Nevertheless, the potential mechanism of *L. rhamnosus* in the amelioration of acne vulgaris has not been elucidated yet.

**Methods:**

To mimic the human intestinal environment, a pseudo-germ-free rat model was used, and then gut microbiota from healthy individuals and acne patients were transplanted into rats. The effects of *L. rhamnosus* and tryptophan (Trp) metabolites on a rat acne model were investigated by gavage. Then, 16S rRNA analysis and targeted measurement of metabolites were performed to discover the differences in gut microbiota and metabolites between groups. Finally, HaCaT cells pretreated with *Cutibacterium acnes* were employed to validate the effect and mechanism of Trp metabolites on acne.

**Results:**

*L. rhamnosus* significantly improved acne-like symptoms in rats by suppressing the level of inflammatory cytokines such as *IL-1β*, *IL-6*, and *TNF-α*. *L. rhamnosus* induced an increase in the production of indole-3-acetic acid (IAA) and indole *via* targeted Trp metabolic analyses. Furthermore, *L. rhamnosus* promoted bacterial diversity and also enhanced the Firmicutes/Bacteroidota (F/B) ratio, which was positively related to both IAA and indole. Finally, the roles of IAA and indole in alleviating acne vulgaris were confirmed both *in vitro* and *in vivo*, which could be reversed by AhR inhibitors.

**Conclusion:**

Our study demonstrated that *L. rhamnosus* could exert its therapeutic effects on acne vulgaris by modulating the gut microbiota and regulating associated Trp metabolites.

## 1 Introduction

Acne vulgaris is a common chronic inflammatory skin disease that affects approximately 85% of adolescents, leading to damaged appearance and even depression, anxiety, and suicidal tendencies in patients. According to the guidelines, treatment intensity depends on the severity of the acne and should be initiated with topical medications, such as adapalene, benzyl peroxide, and clindamycin (1). To treat severe recalcitrant acne vulgaris, systemic isotretinoin and antibiotics have been suggested as the first-line therapy. However, adverse effects including dryness, irritation, redness, peeling, embryotoxic and teratogenic characteristics of isotretinoin, and antibiotic resistance limit their application. Further investigation into safe and effective treatments for acne vulgaris remains of clinical value.

The concept that gut microbiota contribute to the pathogenesis of acne vulgaris originated in the 1980s. However, only a few studies demonstrated the causal relationship between gut microbiota and the development of acne vulgaris (2). Recently, our previous studies and Yan et al. revealed the distinctly disordered gut microbiota in individuals diagnosed with acne vulgaris, compared to healthy people (3–5). The depletion of potentially beneficial taxa such as *Lactobacillus*, *Clostridia*, Ruminococcaceae, and *Bifidobacterium* found in acne patients suggested that probiotic supplementation may be a potential new approach to acne treatment. In fact, a few studies have indicated that the administration of oral probiotics, especially *Lactobacillus rhamnosus*, could lead to the normalization of skin expression of genes involved in insulin signaling and improvement of the appearance of adult acne (6–8). *Lactobacillus rhamnosus* (*L. rhamnosus*) was initially discovered in the gut microbiota of healthy individuals in the 1980s, and it was reported to regulate gut microbiota imbalance, protect intestinal barrier function, reduce inflammation, and modulate intestinal immunity (9–12). However, the specific mechanism of probiotics in treating acne vulgaris is still unknown.

It is acknowledged that gut microbiota frequently exert their effects on distant organs *via* microbiota metabolites, which implies how probiotic supplements work (13). Tryptophan (Trp) is an essential amino acid for the human body that is metabolized by gut commensals, yielding compounds that are involved in many diseases. Changes in Trp metabolites were reported to have a profound impact on the clinical characteristics of patients with depression, food allergies, and cancer (14–16). In an encephalomyelitis mouse model, supplementation with Trp or Trp-derived aryl hydrocarbon receptor (AhR) agonists increased IFN-suppressive effects in an AhR-dependent manner and reduced central nervous system inflammation (17). A study reported that the Trp metabolite indole-3-aldehyde (IAId) has a negative regulatory effect on Langerhans cells, promoting the expression of RANK and RANKL on Langerhans cells and keratinocytes in an AhR-dependent manner, which may result in the activation of NF-κB signaling and production of IL-10 (18). Another study revealed that IAId significantly reduced skin inflammation in mice with MC903-induced AD-like dermatitis, and this effect was blocked by an AhR antagonist and eliminated in AhR-null mice (19).

In combination with these alterations, we hypothesized that *L. rhamnosus* may alleviate acne clinical symptoms by modulating Trp metabolism, and this study aimed to explore 1) the effect of *L. rhamnosus* on acne symptoms and 2) the mechanism of *L. rhamnosus* in alleviating acne vulgaris through gut microbiota and their associated Trp metabolism.

## 2 Materials and methods

### 2.1 *L. rhamnosus and Cutibacterium acnes* strains

*L. rhamnosus* 185356 used in this study was received from Shenzhen Yibaishun Technology Co., Ltd. (Shenzhen, China) and was aerobically cultured in de Man, Rogosa, and Sharpe (MRS) broth at 37°C for 24 to 48 h. The total viable bacteria were counted on the MRS agar plate with serial dilution to ensure the number of viable microorganisms in a given dose.

*C. acnes* (ATCC6919, China) obtained from Guangdong Microbial Culture Center was inoculated in brain heart infusion and incubated at 37°C for 72 h. The concentration of *C. acnes* suspension was prepared at 1 × 10^7^ cells/ml. The suspension was inactivated in a 95°C water bath for 10 min to facilitate the heat-killing reaction and stored at 4°C until further use.

### 2.2 Animals

Three-week-old male Sprague Dawley (SD) rats (65–105 g) were purchased from the Center of Experimental Animals of Southwest Medical University (Sichuan, China). The rats were housed in a specific pathogen-free (SPF) laboratory environment with constant temperature, humidity, and a 12-h light/dark cycle. All the animal experiments were performed in accordance with the Guide for the Care and Use of Laboratory Animals and approved by the Ethical Management Committee of Animal Experimentation of the Affiliated Hospital of Southwest Medical University (approval no. swmu20220216).

### 2.3 Preparation for fecal microbiota transplantation

Stool samples were collected from patients with acne and healthy people and immediately stored at −80°C. Pooled frozen stool samples at a volume of 30 g were ground using a mortar and pestle and added to 150 ml of sterile normal saline. After mixing, the samples were filtered through 2.0, 1.0-mm, 0.5-mm, and 0.25-mm stainless steel screens, followed by centrifugation at 6,000 *g* for 15 min. The remaining material was homogenized in 150 ml of sterile saline. It was diluted to a final concentration of 10% by adding 17 ml of glycerol (99%) and then quickly stored at −80°C for further use (completed within 2 h). All equipment used in the fecal suspension preparation was strictly sterile. To ensure the activity of fecal microorganisms, oxygen exposure time was minimized when preparing fecal materials. All fecal materials were prepared at room temperature of 20°C–30°C, preferably in an anaerobic incubator.

### 2.4 Animal experimental design

In research 1, SD rats were applied after 1 week of adaptive feeding. All rats were divided into three groups and received antibiotics (ampicillin sodium 1 g/L, neomycin sulfate 1 g/L, and metronidazole 0.5 g/L) intragastrically once a day for 14 days to deplete the gut microbiota before acne modeling and fecal microbiota transplantation (FMT). Then, SD rats were treated with saline (acne group) or transplanted with gut microbiota from healthy people (NM-FMT group) or acne patients’ fecal bacteria (AC-FMT group) by gavage once a day for another 7 days and then once every other day for 14 days. The acne model was established 7 days after gavage, with 0.3 ml oleic acid evenly applied to the right auricle of the rats once a day and *C. acnes* suspension (50 μl/200 g) intradermally injected once every other day for 14 days. The 0.3 ml normal saline was evenly applied to the right auricle of another six rats for 14 days as the control group, which also received saline gavage.

In research 2, all rats were randomly divided into three groups: NM-FMT group, AC-FMT group, and AC-FMT+*L* group. After antibiotic treatment, acne modeling, and FMT, the AC-FMT+*L* group was treated with *L. rhamnosus* gavage simultaneously. The other two groups were the same as above.

In research 3, rats were randomly divided into six groups: control group, acne group, indole-3-acetic acid (IAA) group, indole group (oral administration for 1 mg/100 g once daily for 7 days before acne modeling and then once every other day for another 7 days) (20), IAA+CH group, and indole+CH group (intraperitoneal injection of CH223191). Acne modeling was the same as above. IAA and indole were dissolved in 0.2% sodium carboxymethylcellulose and 0.25% polysorbate-80 in phosphate-buffered saline (PBS). CH223191 (an AhR antagonist, Sigma-Aldrich, Steinheim, Germany) was dissolved in 20 L of 10% dimethyl sulfoxide (DMSO), and then 1 ml of corn oil was added, followed by ultrasonic vibration at 70 Hz repeated three times for 15 seconds prior to intraperitoneal injection.

### 2.5 Evaluation of acne-like lesions in rats

Rats’ auricle alterations were captured on camera before and after acne modeling, and the severity of skin lesions was evaluated by measuring the swelling rate of the auricle.

### 2.6 Evaluation of pseudo-germ-free rat model

The changes in gut microbiota diversity (Chao index) before and after antibiotic treatment were detected to evaluate whether the pseudo-germ-free model was successfully constructed.

### 2.7 Hematoxylin and eosin and immunohistochemistry

Ear tissue was taken and immediately preserved in 4% paraformaldehyde for 6–8 h, and after gradient dehydration, samples were cut into 3-μm-thick serial sections for hematoxylin and eosin (H&E) staining. The sections were observed under a microscope (Olympus, IX73-F22PH, Tokyo, Japan), and the mean number of microcomedo and single nucleated cells in each group of rat auricular tissue sections was counted in three random fields of view at ×100 and ×400, respectively.

Auricle tissues were incubated in ethylenediaminetetraacetic acid (EDTA) repair solution at 95°C for 20 min and then cooled to room temperature. Sections were washed and incubated in endogenous peroxidase blocker for 20 min to quench endogenous peroxidase activity and then closed with 10% bovine serum albumin (BSA) in PBS at room temperature for 2 h. Sections were then incubated with *TNF-α*, *IL-1β*, and *IL-1α* antibodies (1:50) overnight at 4°C, followed by the addition of enzyme-labeled goat anti-mouse/rabbit IgG polymer dropwise onto the tissue of slides. Specific labeling was performed using the diaminobenzidine substrate kit, and specimens were restained with hematoxylin and observed under a microscope.

### 2.8 Liquid chromatography–tandem mass spectrometry

The fecal samples of SD rats were collected on 0 days and 15 days and rapidly stored at −80°C. Targeted metabolomics was used to identify the major metabolites of the intestinal Trp catabolic pathway. Liquid chromatography–tandem mass spectrometry (LC-MS/MS) data were acquired on AB SCIEX 5500 QQQ mass spectrometer (Applied Biosystems, Foster City, CA, USA) coupled with high-performance liquid chromatography (HPLC) system ACQUITY UPLC (Waters, Milford, MA, USA). The raw mass spectrometry data were analyzed using MultiQuant software.

### 2.9 Integration of metagenomes and metabolomes

Sequences were clustered into operational taxonomic units (OTUs). The OTUs that reached 97% nucleotide similarity level were used for further analysis. According to the 97% similarity to a repetitive sequence (not including a single sequence), Uparse software (v7.0.1090, http://drive5.com/uparse/) was adopted to improve the OTU clustering. Phylogenetic investigation of communities by reconstruction of unobserved states (PICRUSt) was used to infer metagenomes based on the 16S marker data and predicted Kyoto Encyclopedia of Genes and Genomes (KEGG) pathway abundances on the free online platform Majorbio Cloud Platform (http://www.majorbio.com).

### 2.10 Cell culture and intervention

Human keratinocyte cells (HaCaT cell line) were purchased from the MeisenCTCC (CTCC-002-0012; Pan’an, Zhejiang, China). HaCaT cells were cultured in high-glucose Dulbecco’s modified Eagle’s medium (DMEM) (Gibco, Life Technologies, Carlsbad, CA, USA) containing 10% (v/v) fetal bovine serum (FBS; Every Green, Tianhang, Zhejiang, China) and 1% (v/v) penicillin/streptomycin (pen/strep; 100 UI/ml, Sigma-Aldrich, Steinheim, Germany) and maintained in a humidified atmosphere at 37°C with 5% CO_2_.

HaCaT cells were pre-exposed to 10^7^ CFU/ml, 10^8^ CFU/ml, or 10^9^ CFU/ml of heat-killed *C. acnes* for 24 h and then co-cultured with IAA and indole (purity > 99%; Macklin, Shanghai, China) for another 24 h, which were dissolved in DMSO before use. Meanwhile, the AhR inhibitor StemRegenin 1 (SR1, MedChemExpress, Shanghai, China) was used to treat HaCaT cells at a concentration of 100 nM for 24 h. Cell Counting Kit-8 (CCK-8; Bao Guang, Chongqing, China) was applied to quantify cell viability, and the absorbance values at 450 nm were determined using a microplate reader.

### 2.11 qRT-PCR

Total RNA was extracted from HaCaT according to the manufacturer’s specifications using the RNAsimple Total RNA Kit (TIANGEN, Beijing, China). The mRNA was dissolved in DEPC water and quantified using the NanoDrop ND-2000 spectrophotometer (Thermo Fisher Scientific, Waltham, MA, USA). Reverse transcription was performed using the ReverTra Ace qPCR RT Master Mix Kit (TOYOBO, Osaka, Japan). SYBR Green Realtime PCR Master mixture was added to the primers and cDNA in a 96-well PCR plate in triplicate. β-Actin was used as the control gene. The primer sequences are summarized in **Supplementary Table 1**.

### 2.12 Statistical analysis

All experiments were conducted in triplicate and analyzed using a non-parametric test using GraphPad Prism version 8.0 software. ImageJ software was used for the analysis of immunohistochemistry data. Wilcoxon rank-sum test was used to identify the difference between the diversity index, and the Kruskal–Wallis rank-sum test was used to screen the differential bacteria combined with the multiple testing method false discovery rate (FDR). All plots are shown as mean ± SEM. *p*-Value <0.05 was considered statistically significant. Images were created using Adobe Illustrator 2021.

## 3 Results

### 3.1 *L. rhamnosus* pathologically alleviated acne-like skin lesions and inhibited inflammation of auricles in rats

After the pseudo-germ-free model was successfully constructed (**Supplementary Figure 1**), this study verified the causal relationship between gut microbiota and the development of acne vulgaris in rat models by FMT. It showed that rats transplanted with gut microbiota from acne patients (AC-FMT) demonstrated more severe clinical and pathological symptoms, compared with those transplanted with gut microbiota from healthy individuals (NM-FMT). Interestingly, the AC-FMT group showed a significantly lower abundance of *Lactobacillus* (*p* = 0.028) than the NM-FMT group. Through differential bacterial analysis of the gut microbiota of SD rats, we discovered that *Lactobacillus* had the most significant difference between the AC-FMT group and the NM-FMT group. The results are discussed in detail in **Supplementary Figure 2**.

**Figure 1:**
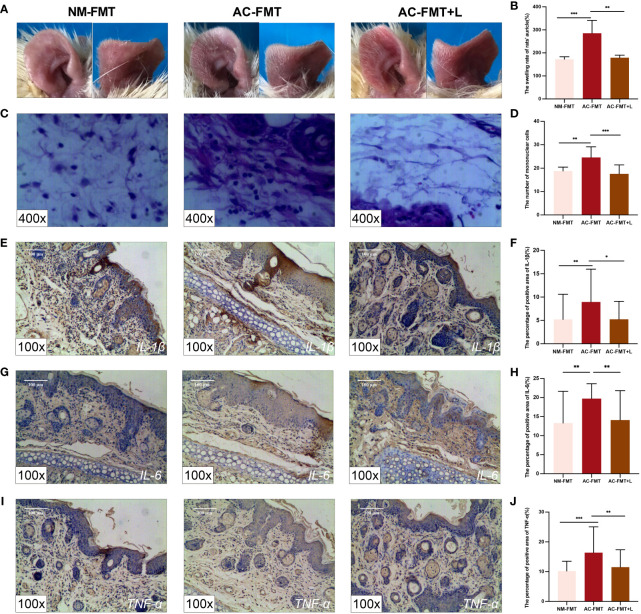
*Lactobacillus rhamnosus* intervention attenuates pathological symptoms and manifestations and inflammation of acne-like lesions in SD rats. **(A)** Auricle lesions of acne modeling in rats (n = 6). **(B)** Comparison of the swelling rate of the auricle (%) in each group (n = 6). **(C)** Histopathological changes in the auricle of rat (n = 6) (H&E staining, ×400). **(D)** Comparison of the number of mononuclear cells (n = 6). **(E)** Expression of *IL-1β* in the auricular tissue of rats (×100). **(F)** Comparison of the percentage of positive area (%) of *IL-1β*. **(G)** Expression of *IL-6* in the auricular tissue of rats (×100). **(H)** Comparison of the percentage of positive area (%) of *IL-6*. **(I)** Expression of *TNF-α* in the auricular tissue of rats (×100). **(J)** Comparison of the percentage of positive area (%) of *TNF-α*. NM-FMT: administration of fecal microbiota from healthy individuals. AC-FMT: administration of fecal bacteria from acne patients. AC-FMT+*L*: AC-FMT and *L. rhamnosus* treatment. Error bars show means ± SEM. ^*^ *p* < 0.050, ^**^ *p* < 0.010, ^***^ *p* < 0.001. SD, Sprague Dawley.

To further explore whether *Lactobacillus* plays a vital role in inhibiting the progression of acne vulgaris, this study employed *L. rhamnosus* to treat acne in a rat model. All rats were transplanted with gut microbiota from healthy people and patients with acne vulgaris to mimic the human gut. Obviously, the severity of inflammation of the auricular skin was significantly increased in the AC-FMT group, compared to the NM-FMT group. After gavage administration of *L. rhamnosus*, the redness and swelling of the ear lesions in the rat model were significantly reduced (**Figure 1A**).

The quantified comparison of the swelling rate of auricles is shown in **Figure 1B**. Compared with the NM-FMT group (172.400 ± 10.680), the swelling rate of the auricle in the AC-FMT group (285.600 ± 55.780) significantly increased (*p* = 0.0007); when *L. rhamnosus* was used, the ear swelling rate in the AC-FMT+*L* group (179.300 ± 10.950) was significantly decreased (*p* = 0.0016).

The histological changes of the auricle of rats in each group after modeling were observed under a microscope at ×400. As shown in **Figures 1C, D**, the AC-FMT group showed a large number of inflammatory cell infiltration (24.580 ± 4.522) compared to the NM-FMT group (18.790 ± 1.672, *p* = 0.0008). In contrast, less inflammatory cell infiltration (17.570 ± 3.797, *p* = 0.0006) was observed in the AC-FMT+*L* group than in the AC-FMT group. Meanwhile, pathological changes under a microscope at ×100 and microcomedo also displayed a similar tendency as inflammatory cells, as shown in **Supplementary Figure 3**.

To evaluate the immunoregulatory effects of *L. rhamnosus* treatments on rats, the expression of *IL-1β*, *IL-6*, and *TNF-α* was assessed by immunohistochemistry. As is shown in **Figures 1E–J**, the expression of *IL-1β* (8.948 ± 7.025), *IL-6* (19.720 ± 3.911), and *TNF-α* (16.380 ± 8.467) in the AC-FMT group was significantly higher than that in the counterparts (5.213 ± 5.382, 13.300 ± 8.346, and 10.200 ± 3.288, respectively) in the NM-FMT group (*p* = 0.0056, *p* = 0.0048, and *p* = 0.0003, respectively). When the administration of *L. rhamnosus* was employed, the concentrations of the above factors in the AC-FMT+*L* group (5.233 ± 3.849, 14.080 ± 7.742, and 11.520 ± 5.834) remarkably declined (*p* = 0.0414, *p* = 0.0013, and *p* = 0.0051), compared with those in the AC-FMT group.

### 3.2 *L. rhamnosus* improved the diversity of gut microbiota

**Figure 2:**
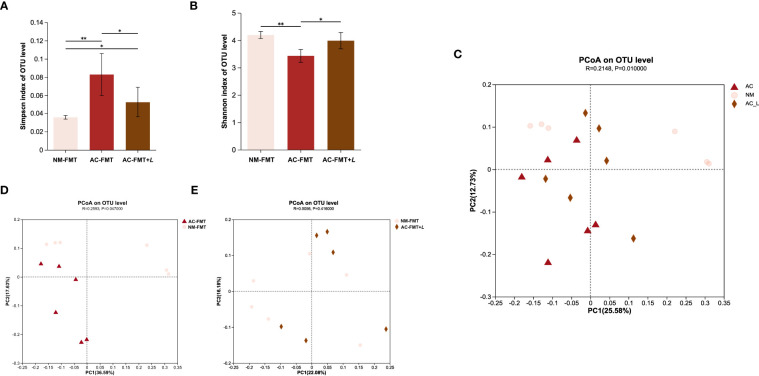
*Lactobacillus rhamnosus* regulates the diversity of gut microbiota in rats. **(A)** Simpson index of OTU level in each group (n = 6). **(B)** Shannon index of OTU level in each group (n = 6). **(C)** PCoA between AC-FMT group and NM-FMT group (n = 6). **(D)** PCoA between NM-FMT group and AC-FMT+*L* group (n = 6). NM-FMT: administration of fecal microbiota from healthy individuals. AC-FMT: administration of fecal bacteria from acne patients. AC-FMT+*L*: AC-FMT and *L. rhamnosus* treatment. Error bars show means ± SEM. ^*^ *p* < 0.050, ^**^ *p* < 0.010. OTU, operational taxonomic unit; PCoA, principal coordinates analysis.

Using the Simpson index and Shannon index to evaluate the richness and diversity of the microbial community after *L. rhamnosus* treatment, as can be seen in **Figures 2A, B**, the AC-FMT group showed less diversity of gut microbiota calculated as Simpson index (0.083 ± 0.023) and Shannon index (3.429 ± 0.234) than the NM-FMT group (0.035 ± 0.002 and 4.193 ± 0.128, respectively) (*p* = 0.005). Thus, the Simpson index (0.053 ± 0.016) significantly decreased and the Shannon index (3.985 ± 0.298) increased in the AC-FMT+*L* group, compared with the AC-FMT group (*p* = 0.045 and *p* = 0.013), after *L. rhamnosus* administration.

Principal coordinates analysis (PCoA) was performed to provide a comprehensive understanding of the similarities and differences between groups. The results showed significant differences in the microbial communities in the AC-FMT, NM-FMT, and AC-FMT+*L* groups (*p* = 0.01) (**Figure 2C**). There was a significant difference in the gut microbial composition between the AC-FMT group and the NM-FMT group (*p* = 0.047), suggesting distinct community constitutions between the two groups (**Figure 2D**). However, after *L. rhamnosus* intervention, there was a close clustering between the AC-FMT+*L* group and NM-FMT group (*p* = 0.416) (**Figure 2E**), which indicated that after *L. rhamnosus* treatment, the gut microbiota in SD rats with acne modeling tended to be restored to normal.

### 3.3 *L. rhamnosus* modified the composition of gut microbiota

**Figure 3:**
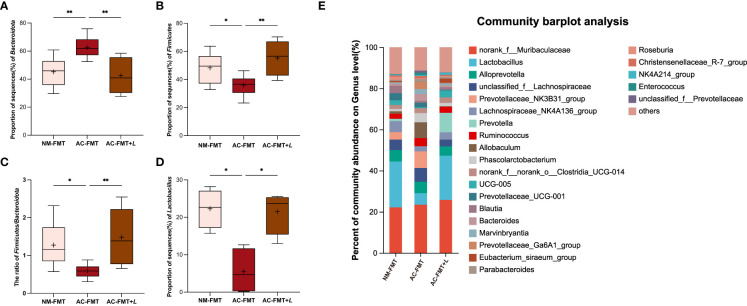
*Lactobacillus rhamnosus* regulates the composition of gut microbiota in rats. **(A)** Proportion of sequence (%) of Bacteroidota (n = 8). **(B)** Proportion of sequence (%) of Firmicutes (n = 8). **(C)** Proportion of sequence (%) of *F/B* ratio (n = 8). **(D)** Proportion of sequence (%) of *Lactobacillus* (n = 4). **(E)** Percent of community abundance at genus level in groups (n = 6). NM-FMT: administration of fecal microbiota from healthy individuals. AC-FMT: administration of fecal bacteria from acne patients. AC-FMT+*L*: AC-FMT and *L. rhamnosus* treatment. Error bars show means ± SEM. ^*^ *p* < 0.050, ^**^ *p* < 0.010.

Next, we analyzed and compared the bacterial changes at various levels in each group. At the phylum level, the abundance of Bacteroidota was significantly higher and that of Firmicutes was less in the AC-FMT group than in the NM-FMT group (*p* = 0.0052 and *p* = 0.0469, respectively). After *L. rhamnosus* intervention, the proportion of Bacteroidota in the AC-FMT+*L* group remarkably decreased and Firmicutes increased when compared with those in the AC-FMT group (*p* = 0.0015 and *p* = 0.0021) (**Figures 3A, B**). As shown in **Figure 3C**, the ratio of Firmicutes/Bacteroidota (*F*/*B*) in the AC-FMT group was less than that in the NM-FMT group (*p* = 0.0130), while the ratio significantly increased in the AC-FMT+*L* group after *L. rhamnosus* treatment, when compared with that in the AC-FMT group (*p* = 0.0053).

At the genus level, it was found that the abundance of *Lactobacillus*, *Prevotella*, *Lachnospiraceae_NK4A136_group*, *Clostridia_UCG-014*, *UCG-005*, *Blautia*, *Eubacterium_siraeum_group*, and *Roseburia* increased in the AC-FMT+*L* group, compared with that in the AC-FMT group (**Figure 3E**). Among them, the increase of *Lactobacillus* was statistically significant (*p* < 0.05). **Figure 3D** shows the change of *Lactobacillus* in each group. Compared with the NM-FMT group, the AC-FMT group had a significant reduction of *Lactobacillus* (*p* = 0.0372). After the gavage of *L. rhamnosus*, *Lactobacillus* had a sharp increase in the AC-FMT+*L* group, when compared with the AC-FMT group (*p* = 0.0372).

### 3.4 *L. rhamnosus* affects the intestinal Trp metabolism and related derived metabolites

**Figure 4:**
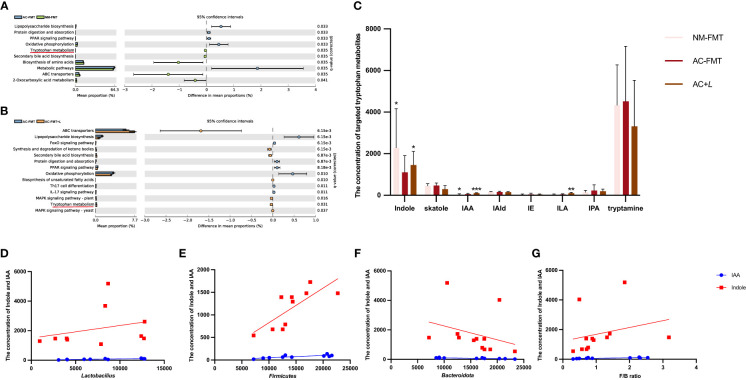
*Lactobacillus rhamnosus* affects intestinal tryptophan metabolism and related derived metabolites. **(A)** Significantly different functional pathways between AC-FMT group and NM-FMT group. **(B)** Significantly different functional pathways between AC-FMT group and AC-FMT+*L* group. **(C)** LC/MS analysis of targeted tryptophan metabolite levels in feces from rats (n = 6). **(D)** Correlation analysis between indole, IAA, and *Lactobacillus*. **(E)** Correlation analysis between indole, IAA, and Bacteroidota. **(F)** Correlation analysis between indole, IAA, and Firmicutes. **(G)** Correlation analysis between indole, IAA, and F/B ratio. NM-FMT: administration of fecal microbiota from healthy individuals. AC-FMT: administration of fecal bacteria from acne patients. AC-FMT+*L*: AC-FMT and *L. rhamnosus* treatment. Error bars show means ± SEM. ^*^ *p* < 0.050, ^**^ *p* < 0.010, ^***^ *p* < 0.001. IAA, indole-3-acetic acid.

Moreover, the KEGG pathway of PICRUSt2 was used to explore the functional features of the gut microbiota influenced by *L. rhamnosus*. As shown in **Figures 4A, B**, the top 10 functional pathways with significant differences between the AC-FMT and NM-FMT groups and between the AC-FMT and AC-FMT+*L* groups were screened. Compared with those in the NM-FMT group, the pathways of lipopolysaccharide (LPS) biosynthesis significantly increased (*p* = 0.033), while Trp metabolism decreased (*p* = 0.035) in the AC-FMT group. After *L. rhamnosus* intervention, the pathway of Trp metabolism was upregulated and LPS biosynthesis was downregulated when compared with those in the AC-FMT group (*p* = 0.031 and *p* < 0.01, respectively) (**Figure 4B**).

Because *L. rhamnosus* induced the modification of microbial Trp metabolism, the next attempt was to investigate the changes in Trp metabolites associated with gut microbiota through targeted fecal Trp metabolite analysis. Trp metabolites such as indole, IAA, skatole, IAId, indole ethanol, indolepropionic acid, tryptamine, and indole-3-lactate (ILA) in rat feces of all the above three groups were detected. As shown in **Figure 4C**, we can see that there were significant differences in indole, IAA, and ILA among the groups. The concentrations of indole (933.80 ± 545.60) and IAA (40.65 ± 15.56) in the feces of the AC-FMT group were significantly lower than those (2,952.00 ± 1,568.00, 65.11 ± 20.26) in the NM-FMT group (*p* = 0.0152 and *p* = 0.0260, respectively), while they were significantly increased (1,958.00 ± 435.10 and 102.70 ± 19.51, respectively) after *L. rhamnosus* intervention, compared to those in the AC-FMT group (*p* = 0.0022). As for ILA, there was a significant increase in the AC-FMT+*L* group (105.00 ± 39.84) compared with the AC-FMT group (46.42 ± 26.19) (*p* = 0.026), but there was no difference in the NM group.

Then, the correlation analysis of indole and IAA with differential bacteria was conducted, as shown in **Figures 4D–G**. It was found that IAA and indole showed significantly positive correlations with *Lactobacillus* (*p* = 0.024, *R* ^2 ^= 0.669; *p* = 0.049, *R* ^2 ^= 0.079), Firmicutes (*p* = 0.006, *R* ^2 ^= 0.633; *p* = 0.010, *R* ^2 ^= 0.582), and *F/B* ratio (*p* = 0.010, *R* ^2 ^= 0.546; *p* = 0.046, *R* ^2 ^= 0.065), but they were negatively correlated with Bacteroidota (*p* = 0.004, *R* ^2 ^= 0.651; *p* = 0.036, *R* ^2^ = 0.065).

### 3.5 Association between the concentrations of serum IAA and indole and acne vulgaris

**Figure 5:**
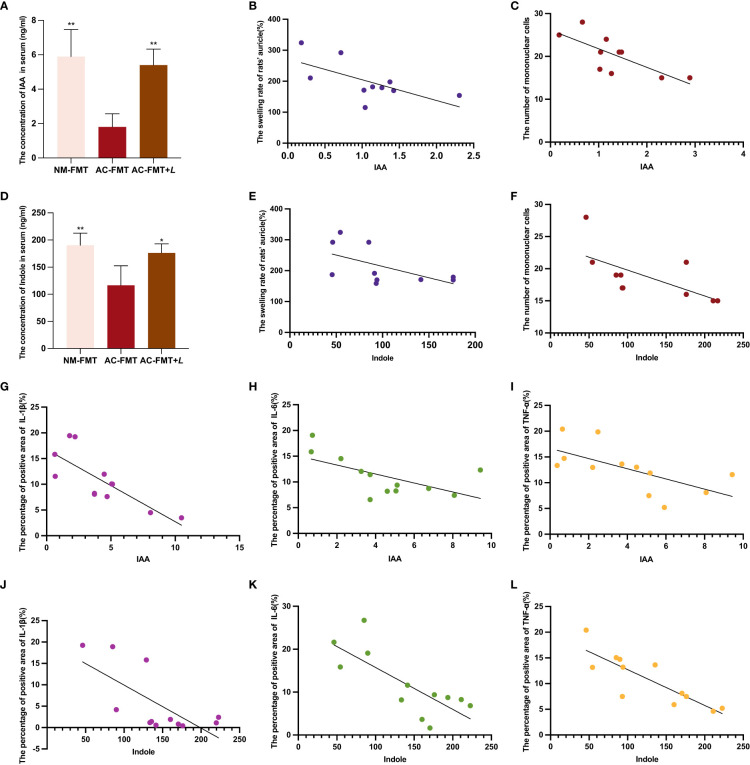
Correlation analysis of serum tryptophan metabolites IAA and indole and acne’s clinical manifestations and inflammatory factors. **(A)** Comparison of the concentration of IAA in serum (n = 4). **(B)** Correlation analysis between IAA and the swelling rate of rats’ auricle (%) (n = 10). **(C)** Correlation analysis between IAA and the number of mononuclear cells (n = 10). **(D)** Comparison of the concentration of indole in serum (n = 4). **(E)** Correlation analysis between indole and the swelling rate of rats’ auricle (%) (n = 10). **(F)** Correlation analysis between indole and the number of mononuclear cells (n = 10). **(G)** Correlation analysis between IAA and the percentage of positive area of IL-1β (n = 12). **(H)** Correlation analysis between IAA and the percentage of positive area of IL-6 (n = 12). **(I)** Correlation analysis between IAA and the percentage of positive area of TNF-α (n = 12). **(J)** Correlation analysis between indole and the percentage of positive area of IL-1β (n = 12). **(K)** Correlation analysis between indole and the percentage of positive area of IL-6 (n = 12). **(L)** Correlation analysis between indole and the percentage of positive area of TNF-α (n = 12). NM-FMT: administration of fecal microbiota from healthy individuals. **(K)** Correlation analysis between indole and the number of mononuclear cells (n = 10). AC-FMT: administration of fecal bacteria from acne patients. AC-FMT+*L*: AC-FMT and *Lactobacillus rhamnosus* treatment. Error bars show means ± SEM. ^*^ *p* < 0.050, ^**^ *p* < 0.010. IAA, indole-3-acetic acid.

In the feces of rats treated with *L. rhamnosus*, two noticeably changed Trp metabolites, IAA and indole, were identified. To validate the change of IAA and indole by *L. rhamnosus* intervention, their serum concentrations were detected by LC-MS. As shown in **Figures 5A, D**, the concentrations of IAA (1.80 ± 0.77) and indole (116.50 ± 35.97) in the serum of the AC-FMT group were significantly lower than those (5.90 ± 1.57 and 190.40 ± 22.26, respectively) in the NM-FMT group (*p* = 0.0017 and *p* = 0.0081, respectively), while they were significantly increased (5.40 ± 0.93 and 176.20 ± 16.77, respectively) after *L. rhamnosus* intervention, compared to those in the AC-FMT group (*p* = 0.0041 and *p* = 0.0258, respectively).

Then, we conducted a correlation analysis of indole and IAA using evaluation indicators of clinical and pathological manifestations of acne, as shown in **Figures 5B, C, E, F**. We found that IAA showed significantly negative correlations with the swelling rate of rats’ auricle (*p* = 0.044, *R* ^2 ^= 0.413) and mononuclear cells (*p* = 0.013, *R* ^2 ^= 0.556), and indole was also negatively correlated with them (*p* = 0.039, *R* ^2 ^= 0.328; *p* = 0.039, *R* ^2 ^= 0.433; *p* = 0.038, *R* ^2 ^= 0.436). As for the inflammatory factors, we discovered that IAA had significant negative correlations with the percentage of the positive area of *IL-1β* (*p* = 0.0170, *R* ^2 ^= 0.4889), *IL-6* (*p* = 0.0330, *R* ^2 ^= 0.3792), and *TNF-α* (*p* = 0.0248, *R* ^2 ^= 0.4103). In the meantime, indole also negatively correlated with the percentage of the positive area of *IL-1β* (*p* = 0.0170, *R* ^2 ^= 0.4889), *IL-6* (*p* = 0.0032, *R* ^2 ^= 0.5969), and *TNF-α* (*p* = 0.0006, *R* ^2 ^= 0.7064) (**Figures 5G–L**).

### 3.6 Trp metabolites IAA and indole attenuated acne vulgaris both *in vivo* and *in vitro*

**Figure 6:**
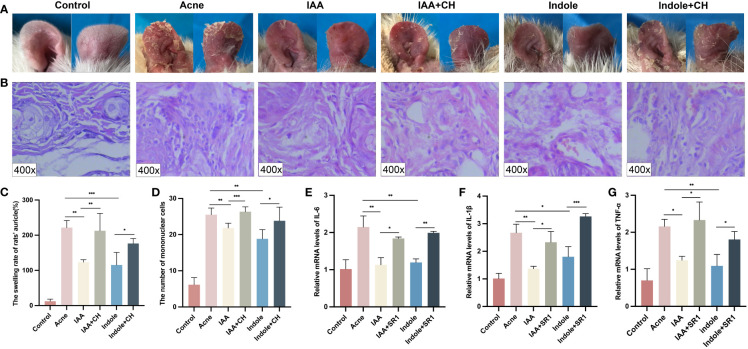
IAA and indole attenuated inflammation in acne compound models both *in vivo* and *in vitro*. **(A)** Changes in auricle lesions after acne modeling in rats (n = 6). **(B)** Histopathological changes in the auricle of rat (n = 6) (H&E staining, ×400). **(C)** Comparison of the swelling rate of the auricle (%) in each group (n = 6). **(D)** Comparison of the number of mononuclear cells (n = 6). **(E)** Comparison of the relative mRNA levels of IL-1β measured by qRT-PCR. **(F)** Comparison of the relative mRNA levels of IL-6 measured by qRT-PCR. **(G)** Comparison of the relative mRNA levels of TNF-α measured by qRT-PCR. Error bars show means ± SEM. ^*^ *p* < 0.050, ^**^ *p* < 0.010, ^***^ *p* < 0.001. IAA, indole-3-acetic acid.

To verify the role of IAA and indole in alleviating acne vulgaris, an acne model of rats was employed, and a combination of AhR antagonists CH223191 as interventions was also used. **Figure 6A** shows that the auricular skin of rats treated with IAA and indole became less red and slightly thickened than that in the acne group, which was reversed by CH223191. Compared with the acne group (221.700 ± 20.130), the swelling rate of the auricle was less severe in the IAA (123.300 ± 7.075, *p* = 0.0027) and indole groups (115.300 ± 35.700, *p* = 0.0027), which also could be aggravated by CH223191 (212.600 ± 49.340 and 176.600 ± 14.070, *p* = 0.0029 and *p* = 0.0203, respectively) (**Figure 6C**).

The histological changes of the auricle of rats in each group after modeling were also observed under a microscope. Both the IAA and indole groups exhibited less epidermal thickening and microcomedo formation in the dermis compared with the acne group as shown in **Supplementary Figure 4A**, which was reversed by CH223191.

The histological changes of the auricle of rats in each group after modeling were observed under a microscope at ×400. As shown in **Figures 6B, D**, the IAA (21.830 ± 1.329) and indole (18.830 ± 2.563) groups exhibited less mononuclear cell infiltration compared with the acne group (25.500 ± 1.871) (*p* = 0.0051 and *p* = 0.0016, respectively), which was reversed by CH223191 (26.330 ± 1.366 and 23.830 ± 3.764, respectively) (*p* = 0.0007 and *p* = 0.0183, respectively).

As for the inflammatory factors, as shown in **Supplementary Figures 4B–G**, the expression of *IL-1β* (6.627 ± 0.473 and 6.667 ± 0.611), *IL-6* (7.067 ± 1.589 and 7.033 ± 0.710), and *TNF-α* (7.433 ± 1.358 and 6.267 ± 0.569) in the IAA group and indole group was significantly decreased than that of their counterparts (11.300 ± 1.153, 13.530 ± 0.982, and 11.470 ± 1.626, respectively) in the acne group (*p* < 0.05). When the administration of CH223191 was employed, the concentrations of *IL-1β* (8.867 ± 1.007 and 9.567 ± 0.289), *IL-6* (11.530 ± 1.701 and 10.900 ± 0.458), and *TNF-α* (11.070 ± 0.950 and 10.400 ± 0.819) were remarkably increased (*p* < 0.05).

To test the role of IAA and indole *in vitro*, HaCaT cells were cocultured with *C. acnes* for 24 h and treated with IAA or indole at a concentration of 500 μM for another 24 h. **Figures 6E–G** demonstrate that the mRNA levels of *IL-1β* (1.368 ± 0.088 and 1.803 ± 0.367), *IL-6* (1.131 ± 0.194 and 1.197 ± 0.952), and *TNF-α* (1.247 ± 0.104 and 1.095 ± 0.306) were significantly lower in the IAA and indole groups, compared to those in the acne group (2.672 ± 0.310, 2.148 ± 0.298, and 2.158 ± 0.193) (*p* < 0.05). All of the above inflammatory factors also could be upregulated by the AhR antagonist in the IAA+SR1 group (2.329 ± 0.386, 1.842 ± 0.037, and 2.331 ± 0.488) and indole+SR1 group (3.265 ± 0.099, 1.991 ± 0.042, and 1.807 ± 0.213) (*p* = 0.0101 and *p* < 0.05).

## 4 Discussion

The objective of this study was to examine the impact of *L. rhamnosus* on the development of acne in rats through the regulation of Trp metabolism in gut microbiota. Microbiota are a key regulator of the immune system, and their primary function is to maintain the internal environment’s homeostasis *via* bidirectional communication with tissues and organs. Scholars have discovered that gut microbiota dysbiosis alters immune response, which plays a pivotal role in many inflammatory skin diseases. Although numerous studies have demonstrated significant differences in gut microbiota between acne patients and healthy controls (3–5), the direct causal link between acne and gut microbiota has not been established. This study demonstrated for the first time that FMT can regulate acne’s clinical and pathological manifestations.

In recent years, therapeutic methods *via* regulating gut microbiota, such as the supplementation of probiotics, have been increasingly used to treat various diseases. This is due to the significant role that gut microbiota serve in the progression of numerous diseases (21–24). The previous study discussed the association between acne vulgaris and changes in gut microbiota and identified that *Lactobacillus* and *Allobaculum* all decreased at the genus level (4). In the meantime, it has been illustrated that supplementation of *L. rhamnosus* SP1 could improve clinical symptoms in acne patients with acne. However, the specific mechanism needs further investigation. This study demonstrated that *L. rhamnosus* could alleviate acne vulgaris by modifying gut microbiota and associated Trp metabolism.

Numerous studies have demonstrated a strong link between the pathogenesis of acne vulgaris and alterations in the composition and diversity of gut microbiota. In our previous research, acne patients showed lower alpha diversity and a decrease in Firmicutes, but an increase in Bacteroidetes at the phylum level (3). In this experiment, the alpha diversity of gut microbiota was significantly elevated by *L. rhamnosus*, as well as the ratio of Firmicutes to Bacteroidetes. By regulating gut microbiota, *L. rhamnosus* can influence other human diseases. Lahtinen et al. (25) discovered that administering *L. rhamnosus* during pregnancy could alter the diversity of fetal intestinal microecology and promote the colonization of specific *Bifidobacterium*. Mendes et al. (26) demonstrated that the addition of *L. rhamnosus* to a high-fat diet increased the diversity of gut microbiota and decreased the proportion of *Proteobacteria*, thereby reducing the leptin resistance caused by a high-fat diet (27). In mice with sepsis, *L. rhamnosus* pretreatment increased intestinal microbial diversity, reshaped the colon microbiome, increased Firmicutes, and decreased *Proteobacteria* and *Deferribacteria* (28).

In this study, we also found that *L. rhamnosus* may treat acne vulgaris by upregulating Trp metabolism. Indole and indole derivatives are catabolites of the microbial Trp metabolism and are associated with immune system maturation and function (29). Previous research has provided evidence indicating that the co-administration of *Lactobacillus plantarum* KLDS 1.0386 and Trp exhibits a protective influence against dextran sulfate sodium (DSS)-induced colitis. Growing evidence suggests that Trp catabolites produced by gut microbiota are important signaling molecules in microbial communities and host-microbial cross-talk and may contribute to intestinal and systemic homeostasis. Probiotics such as *Lactobacillus reuteri* and *Bifidobacterium pseudonidarum* have been reported to produce intestinal Trp metabolites such as IAId and IAA and influence host health (30–32). Our research indicates that *L. rhamnosus* can regulate intestinal Trp to produce indole, IAA, and ILA metabolites and influence acne vulgaris in the host.

As is known, Trp metabolites naturally bind to the AhR receptor to upregulate AhR and reduce inflammation. The underlying mechanism of this protective effect potentially involves the elevation of IAA levels, subsequently leading to the upregulation of AhR expression and the activation of the IL-22/STAT signaling pathway (33). IAA also decreased the incidence and severity of ankylosing spondylitis in mice, inhibited the production of pro-inflammatory cytokines (*TNF-α*, *IL-6*, *IL-17*A, and *IL-23*), increased the production of anti-inflammatory cytokine *IL-10*, and decreased the ratio of pro-inflammatory/anti-inflammatory cytokines (34). In addition, previous research has demonstrated that indole also inhibits TNF-α-mediated NF-κB activation in intestinal epithelial cells, supporting its anti-inflammatory effect (35). In our experiment, we first demonstrated that treatment with IAA and indole may suppress the expression of inflammatory factors in acne vulgaris by the AhR pathway in both *in vivo* and *in vitro*.

This study has a few more limitations that remain unsolved. First, our study is limited to animal and cell experiments, and no human experiments were conducted. Second, it needs to be further confirmed whether IAA and indole are directly produced by *L. rhamnosus*. Nevertheless, our data demonstrated that the concentrations of indole and IAA in the feces and serum of rats were substantially elevated after the gavage of *L. rhamnosus*. In contrast, there is currently no evidence supporting the use of indole for acne treatment; IAA has been used as a photosensitizer in photodynamic therapy for acne (36). In conclusion, *L. rhamnosus* treatment modifies the composition of gut microbiota and enhances microbial Trp metabolism to produce AhR ligands including IAA and indole, thereby activating the AhR signaling pathway and reducing the inflammatory response, offering a novel approach to treating acne vulgaris.

## Data availability statement

The data presented in the study are deposited in the SRA repository, accession number: PRJNA1012678.

## Ethics statement

The animal study was approved by Ethical Management Committee of Animal Experiment of the Affiliated Hospital of Southwest Medical University. The study was conducted in accordance with the local legislation and institutional requirements.

## Author contributions

YuH: Investigation, Writing – original draft, Formal analysis, YaH: Methodology, Project administration. DX: Visualization. LL: Investigation, Validation, Writing – original draft. XX: Funding acquisition, Supervision. YO: Resources. YD: Conceptualization, Funding acquisition, Writing – review & editing.

## Supplementary material

Supplementary Figure 1 Changes in gut microbiota diversity in rats before and after pseudo-germ-free treatment. **(A)** Chao index of OTU level in NM-FMT group (n=3). **(B)** Chao index of OTU level in AC-FMT group (n=3). **(C)** Chao index of OTU level in AC-FMT+*L* group (n=3). NM-FMT: administration with fecal microbiota from healthy human. AC-FMT: administration with fecal bacteria from acne patients. AC-FMT+*L*: AC-FMT and *L. rhamnosus* treatment. Error bars show means ± SEM. **p* < 0.050, ***p* < 0.010, ****p* < 0.001.

Supplementary Figure 2 Transplantation of acne patients or healthy human gut microbiota affects acne lesions in rats. **(A)** Clinical pictures of auricle lesions after acne modeling in rats (n=6). **(B)** Histopathological changes in the auricle of rats (n=6) (HE staining, 100×). **(C)** Histopathological changes in the auricle of rat (n=6) (HE staining, 400×). **(D)** Comparison of the swelling rate of the auricle (%) in each group (n=6). **(E)** Comparison of the number of microcomedo (n=6). **(F)** Comparison of the number of mononuclear cells (n=6). **(G)** Analysis of differential bacteria of gut microbiota in SD rats. Control: blank control. Acne: acne modeling and gavage of normal saline. AC-FMT: acne modeling and administrated with fecal microbiota from acne patients. NM-FMT: acne modeling and administrated with fecal microbiota from healthy people. Error bars show means ± SEM. **p* < 0.050, ***p* < 0.010, ****p* < 0.001.

Supplementary Figure 3
*L. rhamnosus* intervention attenuates pathological symptoms and manifestations and inflammation of acne-like lesions in SD rats. **(A)** Histopathological changes in the auricle of rat (n=6) (HE staining, 100×). **(B)** Comparison of the number of comedones (n=6). NM-FMT: administration with fecal microbiota from healthy humans. AC-FMT: administration with fecal bacteria from acne patients. AC-FMT+*L*: AC-FMT and *L. rhamnosus* treatment. Error bars show means ± SEM. **p* < 0.050, ***p* < 0.010, ****p* < 0.001.

Supplementary Figure 4 IAA and Indole attenuated inflammation in acne compound model *in vivo*. **(A)** Histopathological changes in the auricle of rat (n=6) (HE staining, 100×). **(B)** Expression of *IL-1β* in the auricular tissue of rats (×100). **(C)** Expression of *IL-6* in the auricular tissue of rats (×100). **(D)** Expression of *TNF-α* in the auricular tissue of rats (×100). **(E)** Comparison of the percentage of positive area (%) of *IL-1β* (n=3). **(F)** Comparison of the percentage of positive area (%) of *IL-6* (n=3). **(G)** Comparison of the percentage of positive area (%) of *TNF-α* (n=3). Control: blank control. Acne: acne modeling and gavage of normal saline. IAA: acne modeling and administrated with IAA. IAA+CH: acne modeling and administered with CH223191 by IP injection. Indole: acne modeling and administrated with Indole. Indole+CH: acne modeling and administered with CH223191 by IP injection. Error bars show means ± SEM. **p* < 0.050, ***p* < 0.010, ****p* < 0.001.

## References

[1] Roman CJ Cifu AS Stein SL. Management of acne vulgaris. JAMA (2016) 316(13):1402–3. doi: 10.1001/jama.2016.11842 27701644

[2] Stokes JH Pillsbury DM. The effect on the skin of emotional and nervous states III. Theoretical and practical consideration of a gastro-intestinal mechanism. Arch Dermatol Syphilology (1930) 22(6):962–93. doi: 10.1001/archderm.1930.01440180008002

[3] Deng Y Wang H Zhou J Mou Y Wang G Xiong X. Patients with acne vulgaris have a distinct gut microbiota in comparison with healthy controls. Acta Derm Venereol (2018) 98(8):783–90. doi: 10.2340/00015555-2968 29756631

[4] Yan HM Zhao HJ Guo DY Zhu PQ Zhang CL Jiang W. Gut microbiota alterations in moderate to severe acne vulgaris patients. J Dermatol (2018) 45(10):1166–71. doi: 10.1111/1346-8138.14586 30101990

[5] Huang Y Liu L Chen L Zhou L Xiong X Deng Y. Gender-specific differences in gut microbiota composition associated with microbial metabolites for patients with acne vulgaris. Ann Dermatol (2021) 33(6):531–40. doi: 10.5021/ad.2021.33.6.531 PMC8577912 34858004

[6] Kim J Ko Y Park YK Kim NI Ha WK Cho Y. Dietary effect of lactoferrin-enriched fermented milk on skin surface lipid and clinical improvement of acne vulgaris. Nutrition (2010) 26(9):902–9. doi: 10.1016/j.nut.2010.05.011 20692602

[7] Jung GW Tse JE Guiha I Rao J. Prospective, randomized, open-label trial comparing the safety, efficacy, and tolerability of an acne treatment regimen with and without a probiotic supplement and minocycline in subjects with mild to moderate acne. J Cutan Med Surg (2013) 17(2):114–22. doi: 10.2310/7750.2012.12026 23582165

[8] Fabbrocini G Bertona M Picazo Ó Pareja-Galeano H Monfrecola G Emanuele E. Supplementation with Lactobacillus rhamnosus SP1 normalises skin expression of genes implicated in insulin signalling and improves adult acne. Benef Microbes (2016) 7(5):625–30. doi: 10.3920/bm2016.0089 27596801

[9] Yu J Zhu YH Yang GY Zhang W Zhou D Su JH. Anti-inflammatory capacity of Lactobacillus rhamnosus GG in monophasic variant Salmonella infected piglets is correlated with impeding NLRP6-mediated host inflammatory responses. Vet Microbiol (2017) 210:91–100. doi: 10.1016/j.vetmic.2017.08.008 29103703

[10] Nocerino R Di Costanzo M Bedogni G Cosenza L Maddalena Y Di Scala C. Dietary treatment with extensively hydrolyzed casein formula containing the probiotic lactobacillus rhamnosus GG prevents the occurrence of functional gastrointestinal disorders in children with cow's milk allergy. J Pediatr (2019) 213:137–142 e132. doi: 10.1016/j.jpeds.2019.06.004 31327562

[11] Han SK Shin YJ Lee DY Kim KM Yang SJ Kim DS. Lactobacillus rhamnosus HDB1258 modulates gut microbiota-mediated immune response in mice with or without lipopolysaccharide-induced systemic inflammation. BMC Microbiol (2021) 21(1):146. doi: 10.1186/s12866-021-02192-4 33985438 PMC8120827

[12] Tong L Zhang X Hao H Liu Q Zhou Z Liang X. Lactobacillus rhamnosus GG derived extracellular vesicles modulate gut microbiota and attenuate inflammatory in DSS-induced colitis mice. Nutrients (2021) 13(10):3319. doi: 10.3390/nu13103319 34684320 PMC8541209

[13] Roth W Zadeh K Vekariya R Ge Y Mohamadzadeh M. Tryptophan metabolism and gut-brain homeostasis. Int J Mol Sci (2021) 22(6):2973. doi: 10.3390/ijms22062973 33804088 PMC8000752

[14] Cervenka I Agudelo LZ Ruas JL. Kynurenines: Tryptophan's metabolites in exercise, inflammation, and mental health. Science (2017) 357(6349). doi: 10.1126/science.aaf9794 28751584

[15] Platten M Nollen EAA Rohrig UF Fallarino F Opitz CA. Tryptophan metabolism as a common therapeutic target in cancer, neurodegeneration and beyond. Nat Rev Drug Discovery (2019) 18(5):379–401. doi: 10.1038/s41573-019-0016-5 30760888

[16] Crestani E Harb H Charbonnier LM Leirer J Motsinger-Reif A Rachid R. Untargeted metabolomic profiling identifies disease-specific signatures in food allergy and asthma. J Allergy Clin Immunol (2020) 145(3):897–906. doi: 10.1016/j.jaci.2019.10.014 31669435 PMC7062570

[17] Rothhammer V Mascanfroni ID Bunse L Takenaka MC Kenison JE Mayo L. Type I interferons and microbial metabolites of tryptophan modulate astrocyte activity and central nervous system inflammation via the aryl hydrocarbon receptor. Nat Med (2016) 22(6):586–97. doi: 10.1038/nm.4106 PMC4899206 27158906

[18] Liu X Zhang X Zhang J Luo Y Xu B Ling S. Activation of aryl hydrocarbon receptor in Langerhans cells by a microbial metabolite of tryptophan negatively regulates skin inflammation. J Dermatol Sci (2020) 100(3):192–200. doi: 10.1016/j.jdermsci.2020.10.004 33082071

[19] Yu J Luo Y Zhu Z Zhou Y Sun L Gao J. A tryptophan metabolite of the skin microbiota attenuates inflammation in patients with atopic dermatitis through the aryl hydrocarbon receptor. J Allergy Clin Immunol (2019) 143(6):2108–2119 e2112. doi: 10.1016/j.jaci.2018.11.036 30578876

[20] Zhao C Hu X Bao L Wu K Feng L Qiu M. Aryl hydrocarbon receptor activation by Lactobacillus reuteri tryptophan metabolism alleviates Escherichia coli-induced mastitis in mice. PloS Pathog (2021) 17(7):e1009774. doi: 10.1371/journal.ppat.1009774 34297785 PMC8336809

[21] Dziedzic A Saluk J. Probiotics and commensal gut microbiota as the effective alternative therapy for multiple sclerosis patients treatment. Int J Mol Sci (2022) 23(22):14478. doi: 10.3390/ijms232214478 36430954 PMC9699268

[22] Corrie L Awasthi A Kaur J Vishwas S Gulati M Kaur IP. Interplay of gut microbiota in polycystic ovarian syndrome: role of gut microbiota, mechanistic pathways and potential treatment strategies. Pharm (Basel) (2023) 16(2):197. doi: 10.3390/ph16020197 PMC9967581 37259345

[23] Li J Wang J Wang M Zheng L Cen Q Wang F. Bifidobacterium: a probiotic for the prevention and treatment of depression. Front Microbiol (2023) 14:1174800. doi: 10.3389/fmicb.2023.1174800 37234527 PMC10205982

[24] Manfredini M Sticchi A Lippolis N Pedroni G Giovani M Ciardo S. Characterization of acne-prone skin with reflectance confocal microscopy and optical coherence tomography and modifications induced by topical treatment and probiotic supplementation. J Clin Med (2023) 12(14):4787. doi: 10.3390/jcm12144787 37510902 PMC10381777

[25] Lahtinen SJ Boyle RJ Kivivuori S. Prenatal probiotic administration can influence Bifidobacterium microbiota development in infants at high risk of allergy. J Allergy Clin Immunol (2009) 123(2):499–501. doi: 10.1016/j.jaci.2008.11.034 19135234

[26] Mendes MCS Paulino DS Brambilla SR Camargo JA Persinoti GF Carvalheira JBC. Microbiota modification by probiotic supplementation reduces colitis associated colon cancer in mice. World J Gastroenterol (2018) 24(18):1995–2008. doi: 10.3748/wjg.v24.i18.1995 PMC5949713 29760543

[27] Cheng YC Liu JR. Effect of lactobacillus rhamnosus GG on energy metabolism, leptin resistance, and gut microbiota in mice with diet-induced obesity. Nutrients (2020) 12(9):2557. doi: 10.3390/nu12092557 32846917 PMC7551584

[28] Chen L Li H Chen Y Yang Y. Probiotic Lactobacillus rhamnosus GG reduces mortality of septic mice by modulating gut microbiota composition and metabolic profiles. Nutrition (2020) 78:110863. doi: 10.1016/j.nut.2020.110863 32593948

[29] Agus A Planchais J Sokol H. Gut microbiota regulation of tryptophan metabolism in health and disease. Cell Host Microbe (2018) 23(6):716–24. doi: 10.1016/j.chom.2018.05.003 29902437

[30] Russell WR Duncan SH Scobbie L Duncan G Cantlay L Calder AG. Major phenylpropanoid-derived metabolites in the human gut can arise from microbial fermentation of protein. Mol Nutr Food Res (2013) 57(3):523–35. doi: 10.1002/mnfr.201200594 23349065

[31] Cervantes-Barragan L Chai JN Tianero MD Di Luccia B Ahern PP Merriman J. Lactobacillus reuteri induces gut intraepithelial CD4(+)CD8alphaalpha(+) T cells. Science (2017) 357(6353):806–10. doi: 10.1126/science.aah5825 PMC5687812 28775213

[32] Wilck N Matus MG Kearney SM Olesen SW Forslund K Bartolomaeus H. Salt-responsive gut commensal modulates T(H)17 axis and disease. Nature (2017) 551(7682):585–9. doi: 10.1038/nature24628 PMC6070150 29143823

[33] Shi J Du P Xie Q Wang N Li H Smith EE. Protective effects of tryptophan-catabolizing Lactobacillus plantarum KLDS 1.0386 against dextran sodium sulfate-induced colitis in mice. Food Funct (2020) 11(12):10736–47. doi: 10.1039/d0fo02622k 33231244

[34] Shen J Yang L You K Chen T Su Z Cui Z. Indole-3-acetic acid alters intestinal microbiota and alleviates ankylosing spondylitis in mice. Front Immunol (2022) 13:762580. doi: 10.3389/fimmu.2022.762580 35185872 PMC8854167

[35] Bansal T Alaniz RC Wood TK Jayaraman A. The bacterial signal indole increases epithelial-cell tight-junction resistance and attenuates indicators of inflammation. Proc Natl Acad Sci U.S.A. (2010) 107(1):228–33. doi: 10.1073/pnas.0906112107 PMC2806735 19966295

[36] Na JI Kim SY Kim JH Youn SW Huh CH Park KC. Indole-3-acetic acid: a potential new photosensitizer for photodynamic therapy of acne vulgaris. Lasers Surg Med (2011) 43(3):200–5. doi: 10.1002/lsm.21029 21412803

