# Supplementary material for: Lactobacillus rhamnosus ameliorates acne vulgaris in SD rats via changes in gut microbiota and associated tryptophan metabolism
Source: Front Immunol. 2024 Jan 5;14:1293048. doi: 10.3389/fimmu.2023.1293048 (PMC10796797; doi:10.3389/fimmu.2023.1293048)
Supplement: Supplementary file 1 [file Table_1.docx]

Supplementary Table 1 Primers used to analyze gene expression by RT-qPCR

| Gene | Forward primer（5′→3′） | Reverse primer（5′→3′） |
| --- | --- | --- |
| β-actin | TGGCACCCAGCACAATGAA | CTAAGTCATAGTCCGCCTAGAAGCA |
| IL-1β | GTGGCAATGAGGATGACTTGTTC | TAGTGGTGGTCGGAGATTCGTA |
| IL-6 | AGCCACTCACCTCTTCAGAAC | GCCTCTTTGCTGCTTTCACAC |
| TNF-α | CTGCTGCACTTTGGAGTGAT | AGATGATCTGACTGCCTGGG |
