# Supplementary figures and images for: Lactobacillus rhamnosus ameliorates acne vulgaris in SD rats via changes in gut microbiota and associated tryptophan metabolism
Source: Front Immunol. 2024 Jan 5;14:1293048. doi: 10.3389/fimmu.2023.1293048 (PMC10796797; doi:10.3389/fimmu.2023.1293048)

**A**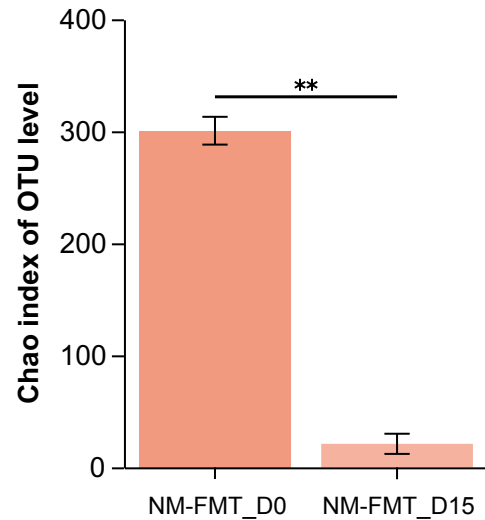**B**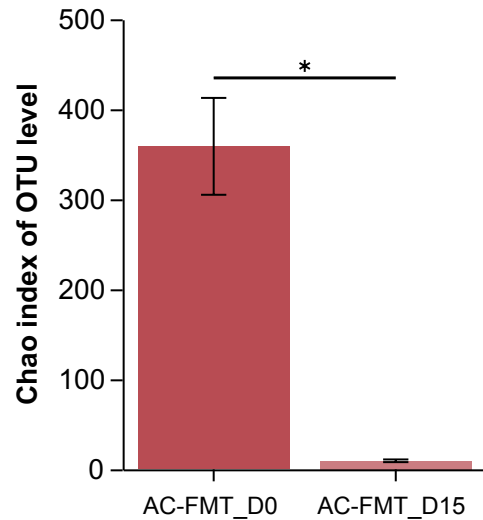**C**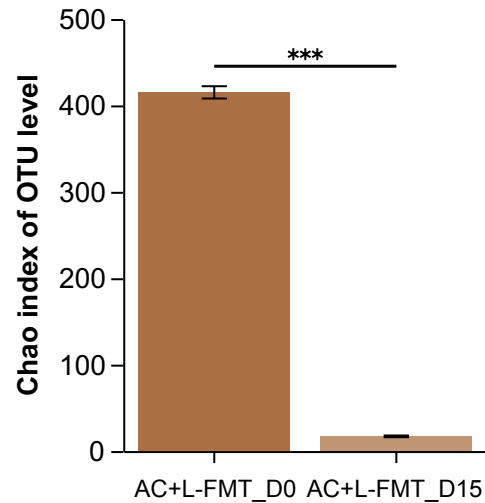

Supplement: Supplementary Figure 1 — Changes in gut microbiota diversity in rats before and after pseudo-germ-free treatment. (A) Chao index of OTU level in NM-FMT group (n=3). (B) Chao index of OTU level in AC-FMT group (n=3). (C) Chao index of OTU level in AC-FMT+L group (n=3). NM-FMT: administration with fecal microbiota from healthy human. AC-FMT: administration with fecal bacteria from acne patients. AC-FMT+L: AC-FMT and L. rhamnosus treatment. Error bars show means ± SEM. *p < 0.050, **p < 0.010, ***p < 0.001. [file Image_1.pdf]

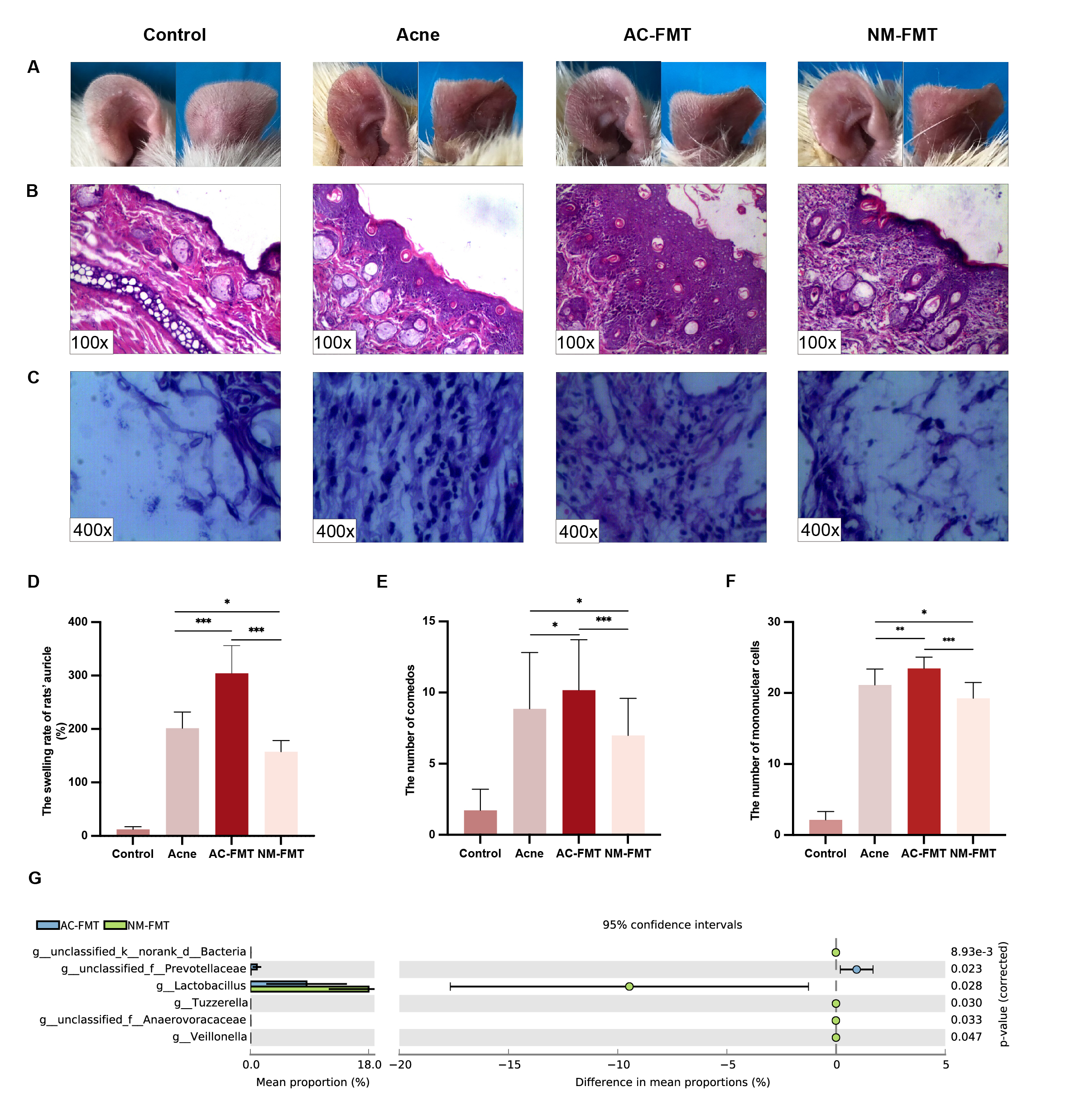

Supplement: Supplementary Figure 2 — Transplantation of acne patients or healthy human gut microbiota affects acne lesions in rats. (A) Clinical pictures of auricle lesions after acne modeling in rats (n=6). (B) Histopathological changes in the auricle of rats (n=6) (HE staining, 100×). (C) Histopathological changes in the auricle of rat (n=6) (HE staining, 400×). (D) Comparison of the swelling rate of the auricle (%) in each group (n=6). (E) Comparison of the number of microcomedo (n=6). (F) Comparison of the number of mononuclear cells (n=6). (G) Analysis of differential bacteria of gut microbiota in SD rats. Control: blank control. Acne: acne modeling and gavage of normal saline. AC-FMT: acne modeling and administrated with fecal microbiota from acne patients. NM-FMT: acne modeling and administrated with fecal microbiota from healthy people. Error bars show means ± SEM. *p < 0.050, **p < 0.010, ***p < 0.001. [file Image_2.jpeg]

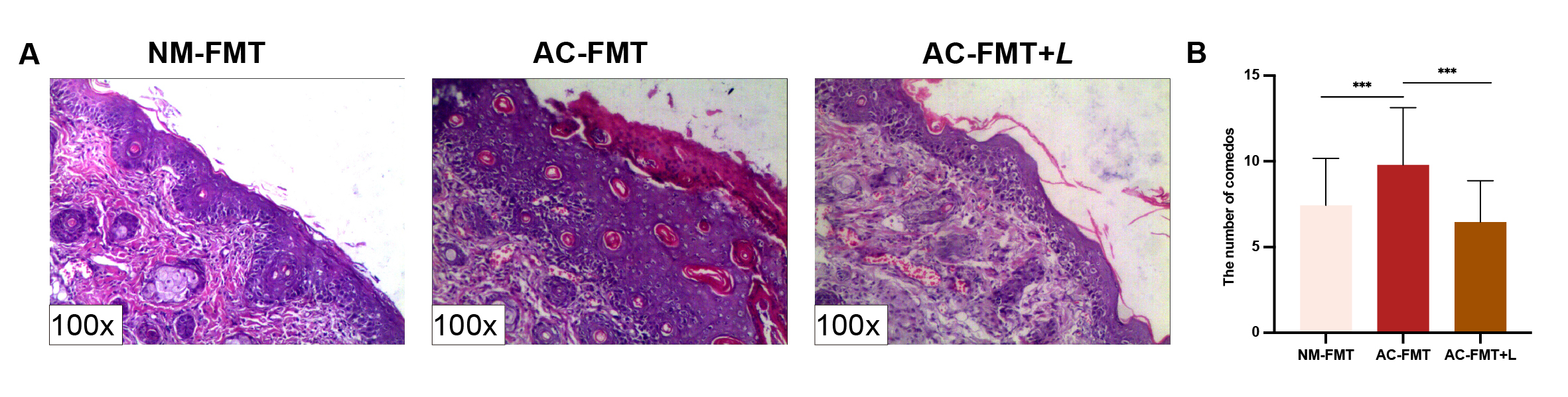

Supplement: Supplementary Figure 3 — L. rhamnosus intervention attenuates pathological symptoms and manifestations and inflammation of acne-like lesions in SD rats. (A) Histopathological changes in the auricle of rat (n=6) (HE staining, 100×). (B) Comparison of the number of comedones (n=6). NM-FMT: administration with fecal microbiota from healthy humans. AC-FMT: administration with fecal bacteria from acne patients. AC-FMT+L: AC-FMT and L. rhamnosus treatment. Error bars show means ± SEM. *p < 0.050, **p < 0.010, ***p < 0.001. [file Image_3.jpeg]

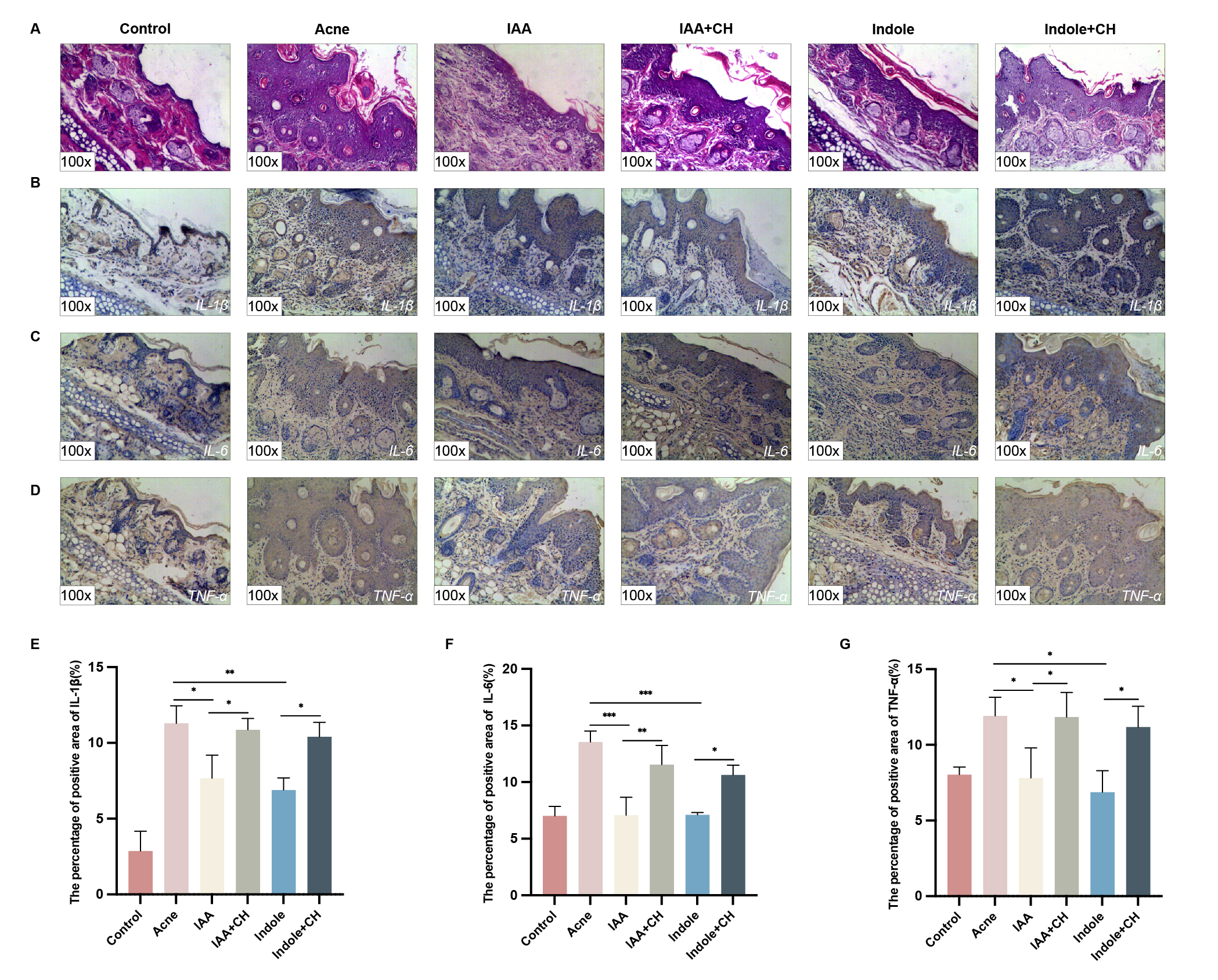

Supplement: Supplementary Figure 4 — IAA and Indole attenuated inflammation in acne compound model in vivo. (A) Histopathological changes in the auricle of rat (n=6) (HE staining, 100×). (B) Expression of IL-1β in the auricular tissue of rats (×100). (C) Expression of IL-6 in the auricular tissue of rats (×100). (D) Expression of TNF-α in the auricular tissue of rats (×100). (E) Comparison of the percentage of positive area (%) of IL-1β (n=3). (F) Comparison of the percentage of positive area (%) of IL-6 (n=3). (G) Comparison of the percentage of positive area (%) of TNF-α (n=3). Control: blank control. Acne: acne modeling and gavage of normal saline. IAA: acne modeling and administrated with IAA. IAA+CH: acne modeling and administered with CH223191 by IP injection. Indole: acne modeling and administrated with Indole. Indole+CH: acne modeling and administered with CH223191 by IP injection. Error bars show means ± SEM. *p < 0.050, **p < 0.010, ***p < 0.001. [file Image_4.jpeg]
